# Retraction: Morpho-physiological and biochemical attributes of Chili (*Capsicum annum* L.) genotypes grown under varying salinity levels

**DOI:** 10.1371/journal.pone.0272532

**Published:** 2022-08-17

**Authors:** The PLOS ONE Editors

The *PLOS ONE* Editors retract this article [1] because it was identified as one of a series of submissions for which we have concerns about authorship, competing interests, and peer review. We regret that the issues were not addressed prior to the article’s publication.

MButt, ASattar, TA, RH, MI, ASher, US, MBrestic, MZ, BSA, AMES, and ATKZ did not agree with the retraction. SU and KG either did not respond directly or could not be reached.
